# Designing eco‐evolutionary experiments for restoration projects: Opportunities and constraints revealed during stickleback introductions

**DOI:** 10.1002/ece3.11503

**Published:** 2024-06-25

**Authors:** Andrew P. Hendry, Rowan D. H. Barrett, Alison M. Bell, Michael A. Bell, Daniel I. Bolnick, Kiyoko M. Gotanda, Grant E. Haines, Åsa J. Lind, Michelle Packer, Catherine L. Peichel, Christopher R. Peterson, Hilary A. Poore, Robert L. Massengill, Kathryn Milligan‐McClellan, Natalie C. Steinel, Sarah Sanderson, Matthew R. Walsh, Jesse N. Weber, Alison M. Derry

**Affiliations:** ^1^ Department of Biology McGill University Montréal Québec Canada; ^2^ School of Integrative Biology University of Illinois at Urbana‐Champaign Urbana Illinois USA; ^3^ Museum of Paleontology University of California Berkeley California USA; ^4^ Department of Ecology and Evolutionary Biology University of Connecticut Storrs Connecticut USA; ^5^ Department of Biological Sciences Brock University Saint Catharines Ontario Canada; ^6^ Aquaculture and Fish Biology Hólar University College Sauðárkrókur Iceland; ^7^ Institute of Ecology and Evolution University of Bern Bern Switzerland; ^8^ Department of Biology University of Texas at Arlington Arlington Texas USA; ^9^ Integrative Biology University of Texas at Austin Austin Texas USA; ^10^ California Energy Commission Sacramento California USA; ^11^ State of Alaska Department of Fish and Game Kalifornsky Alaska USA; ^12^ Department of Molecular and Cell Biology University of Connecticut Storrs Connecticut USA; ^13^ Biological Sciences University of Massachusetts Lowell Lowell Massachusetts USA; ^14^ Integrative Biology University of Wisconsin‐Madison Madison Wisconsin USA; ^15^ Sciences Biologiques Université du Québec á Montréal Montréal Québec Canada

**Keywords:** aquatic ecology, contemporary evolution, ecological dynamics, feedbacks, limnology, rapid evolution

## Abstract

Eco‐evolutionary experiments are typically conducted in semi‐unnatural controlled settings, such as mesocosms; yet inferences about how evolution and ecology interact in the real world would surely benefit from experiments in natural uncontrolled settings. Opportunities for such experiments are rare but do arise in the context of restoration ecology—where different “types” of a given species can be introduced into different “replicate” locations. Designing such experiments requires wrestling with consequential questions. (Q1) Which specific “types” of a focal species should be introduced to the restoration location? (Q2) How many sources of each type should be used—and should they be mixed together? (Q3) Which *specific* source populations should be used? (Q4) Which type(s) or population(s) should be introduced into which restoration sites? We recently grappled with these questions when designing an eco‐evolutionary experiment with threespine stickleback (*Gasterosteus aculeatus*) introduced into nine small lakes and ponds on the Kenai Peninsula in Alaska that required restoration. After considering the options at length, we decided to use benthic versus limnetic ecotypes (Q1) to create a mixed group of colonists from four source populations of each ecotype (Q2), where ecotypes were identified based on trophic morphology (Q3), and were then introduced into nine restoration lakes scaled by lake size (Q4). We hope that outlining the alternatives and resulting choices will make the rationales clear for future studies leveraging our experiment, while also proving useful for investigators considering similar experiments in the future.

## Graphical abstract

Opportunities for eco‐evolutionary experiments are rare but they do arise in the context of restoration ecology. Designing such experiments requires wrestling with several consequential questions. We recently grappled with these questions when designing an eco‐evolutionary experiment with threespine stickleback (*Gasterosteus aculeatus*) introduced into nine small lakes and ponds on the Kenai Peninsula in Alaska that required restoration. We ended up using benthic versus limnetic ecotypes from a mixture of four source populations per ecotype (selected based on trophic morphology) that were then introduced in various combinations into the nine restoration lakes.

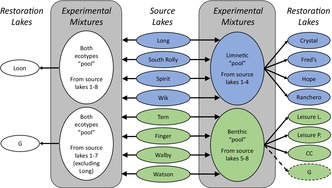

## 1 INTRODUCTION

Evolution was classically considered to be a strictly historical science accessible only indirectly by working backward‐in‐time to infer process from pattern. Increasingly, however, researchers can employ forward‐in‐time studies, where experimental treatments are applied to known populations, and their subsequent evolution is monitored (Garland Jr. & Rose, 2009; Kawecki et al., 2012). Such experiments are commonly conducted in controlled settings, especially laboratories, which allow for high replication and precise control. In an effort to improve realism, similar experiments are sometimes also conducted in semi‐natural field conditions, such as aquatic mesocosms (e.g., cattle tanks or artificial streams), terrestrial cages, or common gardens (Des Roches et al., 2018; Matthews et al., 2011; Rudman et al., 2022). However, the experimental elegance of these (semi)controlled and standardized settings probably renders their outcomes less relevant to the natural world, where noise and confounding influences are always important.

To increase relevance, researchers can conduct evolutionary experiments in completely natural settings, typically by introducing individuals from an ancestral “source” population into new “recipient” environments in nature (Reznick & Ghalambor, 2005). Classic examples of such experiments with vertebrates include planned introductions of Trinidadian guppies (*Poecilia reticulata*) to new predation conditions (Ghalambor et al., 2015; Gordon et al., 2009), *Anolis* lizards to isolated islands (Kolbe et al., 2012; Thurman et al., 2023), and threespine stickleback (*Gasterosteus aculeatus*) to new habitats (Marques et al., 2018). These experiments conducted in nature typically reveal variable outcomes that highlight just how strongly contemporary evolution is shaped by the ecological context of specific experimental sites, the genetic context of specific source populations, and the interaction between these genetic and ecological contexts (Ghalambor et al., 2015; Thurman et al., 2023).

It becomes even more important to conduct experiments in natural settings when the goal is to understand not only evolution, but also the ecological consequences of that evolution—that is, “eco‐evolutionary dynamics” (Hendry, 2017). Specifically, one might argue that we can learn a lot about the nature of *evolution* even in unrealistic ecological settings because the response variables remain embedded in the “natural” genomes of the organism studied. Yet, we clearly cannot learn as much about *ecology* when the response variables are measured in “un‐natural” settings. For example, we might want to understand how the contemporary evolution of different fish ecotypes (e.g., high‐predation vs. low‐predation, benthic vs. limnetic, or stream vs. lake) shapes population dynamics (e.g., abundance, sex ratio, and age structure), community structure (e.g., communities of zooplankton or benthic macroinvertebrates), or ecosystem function (e.g., primary productivity, nutrient cycling, or decomposition). Motivated by such goals, we might place little stock in outcomes from experiments conducted in the laboratory.

Eco‐evolutionary studies thus seek to increase realism by conducting experiments in semi‐natural settings. The typical approach is to place different intraspecific “types” (e.g., different populations, ecotypes, phenotypes, or genotypes) into outdoor common gardens or mesocosms, and to then track changes in various ecological parameters of interest (Matthews et al., 2011). In terrestrial systems, researchers often place individual plants into pots or (somewhat) standardized plots, while sometimes also modifying the surrounding competitors or herbivores. Such experiments typically take place during a single summer, but some with long‐lived trees have continued over many years (Bailey et al., 2009). In aquatic systems, semi‐natural mesocosms variously take the form of stream channels, cattle tanks, ponds dug in the ground, or large plastic “bags” in lakes (Bassar et al., 2012; Matthews et al., 2011; Palkovacs & Post, 2009). These aquatic mesocosm experiments always run for less than a year. In addition to using different intraspecific types, these eco‐evolutionary experiments—whether terrestrial or aquatic—also often include a “species effect” treatment that excludes the focal species altogether or replaces it with a different (but closely related) species.

Meta‐analyses of these mesocosm‐based experiments have revealed surprisingly large ecological effects of intraspecific differences (Des Roches et al., 2018). In particular, the effects of shorter‐term evolutionary differences (i.e., differences within species) are often as large, or sometimes even larger, than the effects of longer‐term evolutionary differences (i.e., differences between species). However, despite these sincere efforts to improve realism through the use of semi‐natural settings, it is safe to say that stream channels are not rivers, cattle tanks are not ponds, plastic bags are not lakes, and common gardens are not forests. As such, it is very hard to know whether these types of experiments are generating inferences that bear any correspondence to the natural environments about which we seek inferences (De Meester et al., 2019; Hendry, 2019). That is, we do not actually care about stream channels, cattle tanks, plastic bags, or common gardens—but we use them because we hope they provide some sort of proxy for what happens in rivers, natural ponds, lakes, or forests. Yet, this hope runs afoul of a large literature showing that spatial scale and experimental venue matter to ecological outcomes For instance, the outcomeof competition, predation, mutualism, and more emergent community dynamics all depend on whether one studies a square meter or a square kilometer plot (Estes et al., 2018) or whether experiments are conducted in the laboratory, mesocosms, or natural ponds (Skelly & Kiesecker, 2001).

Researchers conducting eco‐evolutionary experiments in semi‐natural environments have sought to confirm their relevance to the “real world” in several ways. First, some studies have coupled their empirical findings with parameterized models to infer whether the observed ecological responses correspond to those expected from theory (Bassar et al., 2012). Second, other studies have compared ecological effects in mesocosms to ecological differences between the real systems they are hoped to mimic. For instance, the effects of anadromous (ocean‐going) versus resident (landlocked) alewives (*Alosa pseudoharengus*) on zooplankton communities in mesocosm bags can mirror differences in zooplankton communities between lakes that are naturally exposed to anadromous versus resident alewives (Palkovacs & Post, 2009). Although encouraging, these approaches are not definitive because evolutionary cause and ecological effect in a mesocosm might not correspond to cause and effect in a lake. Stated another way, a comparison between the two contexts might sometimes give the “right” answer (i.e., the same effect) for the “wrong” reason (e.g., a different mechanism).

## 2 ECO‐EVOLUTIONARY EXPERIMENTS IN A RESTORATION CONTEXT

We suggest that *definitive* eco‐evolutionary inferences will sometimes—perhaps often—require experiments conducted in precisely the type of environment about which inferences are desired. For instance, experiments testing the ecological effects of different ecotypes of a lake‐dwelling fish species should be conducted in actual lakes where that species is expected to live, and at the spatial scale of whole lakes. We recognize that such experiments will be impossible or legally/ethically inappropriate in many cases. First, organisms should not be introduced into environments where they are not native or where they can spread to areas where they are not native. Second, organisms should not be introduced into places where populations of that species already exist—although exceptions can occur where such introductions can benefit local populations via demographic, genetic, or evolutionary “rescue” (Carlson et al., 2014) or via “assisted migration” (Twardek et al., 2023). Fortunately, at least one general context exists where eco‐evolutionary introduction experiments are imminently feasible—and, indeed, can be necessary. That context is the restoration of extirpated populations, impoverished communities, or degraded ecosystems (LaRue et al., 2017).

Many freshwater ecosystems are heavily degraded owing to a host of effects that include eutrophication, acidification, pollution, invasive species, overharvesting, damming, and water withdrawals (He et al., 2019; Pérez‐Jvostov et al., 2019). As a result, a number of these ecosystems have seen the disappearance of various fish species. For instance, some whitefish species have disappeared from numerous European lakes (Vonlanthen et al., 2012), and native salmon and trout have been eliminated from hundreds of lakes in North America (Gustafson et al., 2007). Furthermore, the removal of invasive species often involves poisoning with chemicals (e.g., rotenone) that kill all fish, including native species (Pham et al., 2013), as well as lower trophic levels of aquatic food webs (Beaulieu et al., 2021). When such extirpations are widespread, or occur in isolated habitats (e.g., headwater streams or isolated lakes), restoration will require the intentional reintroduction of the missing species. We here ask: How might these conservation‐oriented actions be leveraged to better understand eco‐evolutionary dynamics—and how can eco‐evolutionary considerations improve such restoration efforts?

Once a decision is made to reintroduce a species for restoration, a key question becomes which “type” (ecotype, population, genotype, or ecotype) of that species should be reintroduced (Houde et al., 2015; LaRue et al., 2017; Vergeer et al., 2008)? One reason the decision is not trivial is that different potential source populations can show extensive local adaptation (Hereford, 2009). As a result, the potential pool of colonists might not be well adapted to the intended restoration habitat, especially when that habitat is disturbed (Brady et al., 2019). Furthermore, some source populations could be coincidentally pre‐adapted to the destination habitat, increasing the probability of successful establishment and long‐term population viability (Houde et al., 2015; Vergeer et al., 2008). Other source populations could be more adaptable through plasticity under new conditions or due to more genetic variance upon which selection can act in the new habitat. Indeed, whether to target pre‐adaptation versus evolutionary potential (which might or might not trade‐off with each other) in source material for restoration has been much discussed (e.g., Houde et al., 2015; Vergeer et al., 2008). It is now abundantly clear that the choice of source populations (or ecotypes or phenotypes or genotypes) can be critical to the outcome of reintroduction, and to eco‐evolutionary dynamics in a restoration context.

In most restoration contexts, the optimal “type” of the missing species that should be used as a source for reintroduction is not known. Restoration efforts thus invite—and indeed sometimes require—experiments that place different types of a focal species into different restoration locations. Such experiments then can reveal how variation within a species contributes to evolutionary outcomes, ecological effects, and restoration efforts and goals. Of course, eco‐evolutionary experiments in restoration contexts will have to balance the best design for generating scientific insight with the best design for maximizing immediate restoration benefits. Several core questions come to mind ‐ introduced here and considered further below.

1. *What “types” of the focal species should be introduced?* All species show phenotypic and genetic variation along axes that typically correspond to various selective forces, such as different predators, prey, or parasites. Which of these axes is most useful to leverage in any eco‐evolutionary restoration experiment will depend on factors such as the magnitude of trait variation along each axis, the potential ecological effects of each axis, and the likely selective forces present in the restoration sites. Whatever axis is chosen, one logical approach could be to generate treatments that correspond to the extremes of that axis: for example, high predation versus low predation. Furthermore, the specific axis of variation chosen, and the position of source material along that axis, will depend on whether the goals of the effort are scientific (e.g., to explore the eco‐evolutionary effects of intraspecific variation), practical (e.g., to quickly restore the species or habitat), or a mixture of both.
2. *How many source populations of each “type” should be used, and should they be mixed?* It would seem simplest to introduce one source population of each type (e.g., one high‐predation source population and one low‐predation source population) into multiple recipient (restoration) sites, which thus serve as “replicate” responses to each source population. This two‐source simplicity, however, would introduce several concerns. For instance, robust inferences about the ecological effects of a given source population “type” require replication of that “type”—such as several independent source populations of each type. In this case, it might seem logical to place the different source populations of each type into different restoration sites, yet doing so means that (a) restoration might be severely compromised or delayed if one or more source populations perform poorly, (b) source population will be confounded with restoration lake, and (c) adaptive evolutionary potential might be hampered by limited genetic variation (i.e., that present in a single source population). It therefore makes sense to also consider mixing multiple source populations of each type together (Houde et al., 2015; Vergeer et al., 2008) and introducing each mixture into multiple restoration sites.
3. *Which specific source populations should be used?* Although replication (e.g., different independent populations) of each “type” would be desirable (as explained above), different populations of a given type will not be identical. It is therefore important to survey many candidate source populations and use statistical approaches to decide which specific populations are most representative of (for example) the two extreme types along the evolutionary axis chosen for the experiment. Or perhaps other considerations should also come into play, such as which source populations have the largest population sizes, or are geographically closest to the restoration sites, or are most likely to be independent evolutionary “replicates.”
4. *Which specific source populations should be introduced into which specific restoration sites?* Different restoration sites in the real world will present different conditions for the reintroduced species. It might seem logical that restoration would occur most readily if a given source population were introduced into the “right” environment—that is, the location expected to harbor similar predators or prey or parasites to the site from which the introduced individuals were sourced. Inferentially, however, this simple approach would generate a confound between the experimental treatment and the response environment, which would limit eco‐evolutionary inference. And, of course, intuition about the “right” type of a species for a given restoration site could easily be wrong—most likely due to unrecognized variation in other selective factors. Other options to consider could be random assignment of different source populations to different restoration sites, paired designs that control for some variation among restoration sites, or factorial designs that generate all combinations of colonist types and destination habitats.

These questions are not just academic; they are also practical and real. Whenever reintroductions occur in a conservation effort, managers must make decisions about the choice of source and destination sites. These decisions then have practical effects on researchers' abilities to draw inferences about biological processes. We illustrate these practical considerations by explaining how we confronted them when designing an eco‐evolutionary experiment in a restoration context. By explaining the decisions rendered in our specific situation, we hope to illuminate and exemplify the convergence of experimental design and real‐world practicalities. The present paper thus serves two primary purposes. First, it provides a framework for debating the various issues and opportunities that can arise in such endeavors—and some of the solutions that can be found for mitigating those issues and for leveraging those opportunities. Second, the paper provides context for the core decisions that were made in our specific study, thereby contextualizing all subsequent studies conducted in this large long‐term collaborative enterprise. We start by outlining the specific context for our experiment—and we then turn to the above core questions, the result deliberations, and the solutions on which we settled.

We envision three core audiences for this paper. Most directly, we provide a common basis of understanding for all researchers working on our specific experimental lakes—including external groups that learn of the experiment and would like to test their own scientific ideas. We next hope to inspire ecological and evolutionary researchers working on other systems to consider designing experiments for restoration contexts; and the deliberations we unfold could provide a partial guide to factors those researchers should consider in their own systems. Finally, our work could inspire restoration scientists to add eco‐evolutionary experiments into their project designs. Although our goal is to speak specifically to these three core scientific audiences, it seems possible that managers might also find the paper reassuring when brought to them by collaborating scientists.

## 3 OUR SPECIFIC CONTEXT

The need for restoration—and thus the opportunity for our experiment—arose because an invasive species (the northern pike, *Esox lucius*) was present in several small lakes and ponds on the Kenai Peninsula of Alaska. A related invasive species (the muskellunge, *Esox masquinongy*) was also present in at least one lake. These species had been introduced by anglers because the small lakes lacked water connections to the ocean or any larger water bodies—and, hence, did not naturally contain sport fish species (Dunker et al., 2020).

The pike (but not the muskellunge—details below) soon eliminated native fishes in the lakes, as is often the case when naïve native fishes encounter invasive northern pike (Haught & von Hippel, 2011; Nicholson et al., 2015). Beyond causing local extinctions, invasive pike also dramatically restructure lake food webs (Cathcart et al., 2019). Although the presence of such an invader might be acceptable if it could be contained locally, the chances were deemed high that it might spread (or be spread by people) more widely on the peninsula. The Alaska Department of Fish and Game (ADFG), in consultation with local landowners and communities, thus decided to remove the invasive species and restore the lakes with native fishes.

Pike removal started by angling and gill netting, yet real‐time PCR analyses of genetic markers in water samples indicated that some pike remained. The next step was to use the poison rotenone, which can be very effective at eliminating invasive fishes from lakes (Rytwinski et al., 2019). ADFG performed the rotenone treatment in the lakes we studied in fall 2018 (Couture et al., 2022; Massengill, 2022). The rotenone was successful in eliminating the pike and muskellunge—as confirmed via gill net surveys and by assessing realized rotenone concentrations using both laboratory analysis and caged sentinel fish responses (Dunker et al., 2016; Massengill, 2022). PCR analysis of water samples was not used after rotenone treatment because previous experience by ADFG reveals that pike DNA can be detected for up to several years post‐eradication (Rob Massengill, unpublished data).

Given that the treated lakes did not have open connections to other water bodies, rapid and effective restoration was deemed to require the reintroduction of native fishes. One fish that needed to be reintroduced was the evolutionary model system, the threespine stickleback (*Gasterosteus aculeatus*). At a scientific meeting in summer 2017, Mike Bell explained this upcoming opportunity to Andrew Hendry and Alison Derry, who then asked Rob Massengill of ADFG if the reintroductions could be designed in a manner that would enable the examination of evolutionary and eco‐evolutionary questions in a restoration context. ADFG agreed that such an effort would be beneficial, and so we quickly proceeded to the design and implementation phase.

**FIGURE 1:**
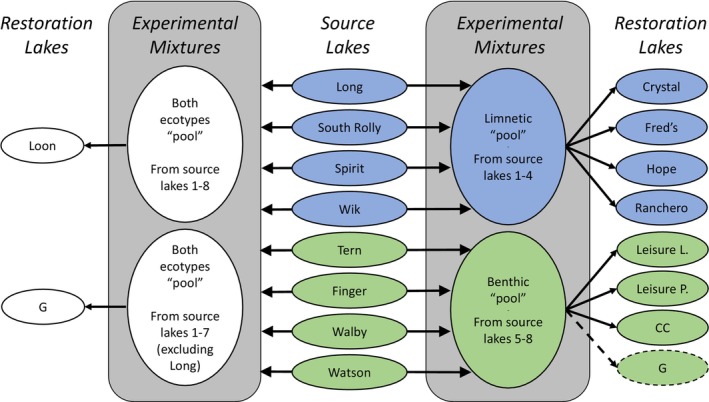
Summary of the final experimental design. The center “Source Lakes” column shows the chosen source lakes and indicates the ecotype of stickleback found within each: blue for limnetic and green for benthic. The gray “Experimental Mixtures” columns then indicate the various mixtures across lakes either within a given ecotype (right gray column) or across both ecotypes (left gray column). The “Restoration Lakes” columns then indicate which restoration lakes received mixtures of a single ecotype (at right) or both ecotypes (at left). Note that G Lake appears on the right as a failed “benthic pool” introduction (indicated with a dashed arrow and oval) in 2019 and on the left as a successful “both ecotypes pool” in 2022.

Here we first briefly outline the *final* design of the experiment so as to provide context for the rest of the narrative. In 2019, (a) stickleback from four source populations of a “limnetic” ecotype were mixed together and introduced into four restoration lakes, (b) stickleback from four source populations of a “benthic” ecotype were mixed together and introduced into four restoration lakes (one of these introductions failed), and (c) stickleback from all eight source populations (both ecotypes) were mixed and introduced into one lake. Then, in 2022, stickleback from seven of the eight source populations were mixed together and introduced into the lake where the benthic ecotype introduction had previously failed (Figure 1). We now unpack the decisions and outcomes that led to this final design—as they provide an example of how theoretical ideals and practical constraints intersect and interact in the design of eco‐evolutionary experiments in restoration contexts.

## 4 Q1. WHAT “TYPES” OF A SPECIES SHOULD BE INTRODUCED?

The standard design of an eco‐evolutionary experiment places two or more types of a given species (e.g., different populations, ecotypes, genotypes, or phenotypes) into replicate experimental arenas and then measures how those different types have different effects on ecological variables of interest (Des Roches et al., 2018; Hendry, 2017). In all such experiments, regardless of venue (laboratory, mesocosm, or nature), an early critical decision is the type of intraspecific variation that should be leveraged to generate different levels of the experimental treatment.

For our experiment with threespine stickleback, several candidate axes of intraspecific variation could be considered, including marine versus freshwater, lake versus stream, high‐calcium water versus low‐calcium water, or benthic versus limnetic (Hendry et al., 2009; McKinnon & Rundle, 2002). We decided to focus on the last of these axes. In lakes, benthic stickleback mainly forage on macroinvertebrates (e.g., chironomids) on the lake bottom, whereas limnetic stickleback mainly forage on zooplankton (e.g., *Daphnia*, copepods) in the open water (Lavin & McPhail, 1985, 1986; Schluter & McPhail, 1992). These designations of “benthic” and “limnetic” are usually not discrete categories, but rather a continuum. Within lakes, individuals vary in their relative reliance on benthic versus limnetic prey; and stable isotope analyses of diets show that most lake populations contain individuals spanning the full range from 0% to 100% benthic prey (100% to 0% limnetic prey), as well as all shades of generalists in between. The exception is a few lakes in British Columbia that contain distinct benthic and limnetic species pairs with few intermediates (Mathews et al., 2010; Schluter & McPhail, 1992). Among lakes, depending on aspects of local lake ecology, the average diets of populations also vary along the benthic‐to‐limnetic axis (Bolnick & Ballare, 2020). The resulting within‐lake and between‐lake continuum of benthic versus limnetic foraging strategies is associated with a suite of differences in trophic traits (Schluter & McPhail, 1992; Willacker et al., 2010). For example, limnetic stickleback have shallower bodies (relative to benthic stickleback), which should be more efficient during sustained swimming in the open water; and they also have more numerous and longer gill rakers, which should facilitate the retention of small prey in the buccal cavity (Lavin & McPhail, 1985, 1986; Schluter & McPhail, 1992).

Our experiment focused on this benthic/limnetic axis of covarying diet and traits for several reasons. First, as introduced above, these foraging differences are a primary axis of intraspecific variation both within and among lakes (Bolnick & Ballare, 2020; Haines et al., 2023; Lavin & McPhail, 1986; Schluter & McPhail, 1992; Willacker et al., 2010). That is, different populations of stickleback, as well as different individuals within populations, are arrayed along this axis between the two dramatic extremes. Second, although the relevant traits can be influenced by developmental plasticity, much of the variation is genetically based (Lavin & McPhail, 1987; Peichel & Marques, 2017). Moreover, researchers have identified some of the important genomic regions, and even specific genes, that shape variation in these traits (Bolnick & Ballare, 2020; Härer et al., 2021; Peichel & Marques, 2017). Third, given the high abundance of stickleback in many lakes (e.g., >75,000 adults in a 112 ha lake: Reimchen, 1990), foraging on benthic versus limnetic prey could be expected to have important—and cascading—ecological consequences. Indeed, experiments in mesocosms have shown that benthic versus limnetic stickleback differentially influence the light environment, zooplankton communities, decomposition rates, and dissolved oxygen concentration (Des Roches et al., 2013; Harmon et al., 2009; Matthews et al., 2016; Rudman & Schluter, 2016). Motivated by this background knowledge, we decided to introduce benthic stickleback into some restoration lakes and limnetic stickleback into other restoration lakes.

Stickleback that are most extreme along the benthic/limnetic axis are probably the sympatric “species pairs” found in a few small lakes in southern British Columbia (Schluter & McPhail, 1992). Using these pairs was not an option in our experiment because they are listed as Endangered under the Canadian Species At Risk Act and, regardless, they are located in another country and far from our study area. Sympatric benthic/limnetic stickleback pairs have not been found elsewhere in the species' range but, fortunately, variation along the same axis is also high among populations in *different* lakes (Lavin & McPhail, 1985, 1986). In particular, lakes that are deeper and more oligotrophic tend to have a prey base dominated by zooplankton, and thus contain stickleback that have evolved a limnetic suite of foraging traits. By contrast, lakes that are shallower and more eutrophic tend to have a prey base dominated by benthic macroinvertebrates, and thus contain stickleback that have evolved a benthic suite of foraging traits. Conveniently, previous work in our study area (Cook Inlet, Alaska) had located and documented some extreme benthic and limnetic populations in different lakes (Willacker et al., 2010). We therefore decided to use benthic and limnetic stickleback from different populations (details below) as the source material for our eco‐evolutionary restoration experiment.

We also considered leveraging variation *within* lakes to further enhance differences between our benthic‐type and limnetic‐type experimental treatments. Indeed, some previous studies of stickleback have performed experiments that selected and compared the limnetic‐most individuals from a lake to the benthic‐most individuals from the same lake (Robinson, 2000). In our case, we could have introduced the benthic‐most individuals from the benthic‐most populations into some restoration lakes and the limnetic‐most individuals from the limnetic‐most populations in other restoration lakes. We decided against this approach for several reasons. First, the effort could be prohibitive as we would have to capture thousands of fish, individually phenotype them, and then only release those individuals showing the most extreme phenotypes. Second, in well‐mixed (i.e., panmictic) populations, even the benthic‐most individuals will carry some limnetic‐type alleles underlying polygenic traits, and the limnetic‐most individuals will carry some benthic‐type alleles. As a result, any selection effort on our part might be quickly diluted by recombination in future generations. Finally, we found an excellent set of contrasting limnetic versus benthic populations (details below) that rendered additional within‐lake sorting unnecessary.

## 5 Q2. HOW MANY SOURCE POPULATIONS OF EACH TYPE SHOULD BE USED, AND SHOULD THEY BE MIXED?

The simplest option was to introduce stickleback from a single benthic source population into some restoration lakes and stickleback from a single limnetic source population into other restoration lakes. We decided against this option for two reasons. First, from a conceptual standpoint, using a single population of each type would lead to a lack of replication (i.e., only one population) of either type, preventing general inferences about the ecological effects of stickleback “type”: that is, benthic versus limnetic. Second, from a pragmatic perspective, using only one source population per ecotype would increase the chances that restoration would fail due to the peculiarities of specific source populations. For example, some populations might be intrinsically less fecund, less capable of phenotypic plasticity to survive in a new habitat, or more heavily infected by pathogens from their native habitat. Specific populations also might be especially susceptible to the stress of capture, processing, or translocation (which proved to be the case ‐ see below).

A second option was to use multiple source populations of each type, with each restoration lake receiving fish from a different source population. For example, restoration lake A could receive stickleback from *benthic source population 1*, restoration lake B could receive stickleback from *benthic source population 2*, restoration lake C could receive stickleback from *limnetic source population 1*, restoration lake D could receive stickleback from *limnetic source population 2*, and so on. We decided against this option because it would completely confound source population and restoration lake, thus eliminating inferences about how outcomes in a given restoration lake might be due to source population or the local environment.

A third option was to break the above confound by, for example, using two source populations of each ecotype, each of which was introduced into two restoration lakes. That is, restoration lakes A and B could receive stickleback from *benthic source population 1*, restoration lakes C and D could receive stickleback from *benthic source population 2*, restoration lakes E and F could receive stickleback from *limnetic source population 1*, and restoration lakes G and H could receive stickleback from *limnetic source population 2*. We decided against this option because the power to uncover effects of source lake type still would be so low (*N* = 2 source populations per type given the nine available lakes) as to represent the worst sort of compromise. Furthermore, placing one source population into each of two restoration lakes would again run the risk of frequent failure due to particular source population peculiarities (as noted above).

The above considerations made clear that the best design would increase the number of source populations, maximize replication of a given treatment across different restoration lakes, and place multiple source populations into each restoration lake. Our solution was to take fish from four source populations of each ecotype and mix them together to create a benthic “pool” of fish (a mixture of four benthic source populations) and a limnetic “pool” of fish (a mixture of four limnetic source populations). Although mixing populations together can lead to outbreeding depression or various other forms of genetic load (Edmands, 1999), we were reassured that this potential problem would not be a major issue in our study because different populations of threespine stickleback are not known to show genetic incompatibilities (Hendry et al., 2009). We next planned for each of the mixed pools to be introduced into four restoration lakes. In this design, each level of the primary “type” treatment (benthic source versus limnetic source) would have four experimental replicates (i.e., each source type would be introduced into four restoration lakes), thus generating reasonable statistical power for inferences about the effects of stickleback “type.”

A limitation of this “mixture” design is that we cannot independently assess the ecological effects of different source populations within a given type—because four populations of each type were always mixed together. Yet, as noted above, alternative designs that allow such inference would have very limited power. Furthermore, an additional set of evolutionary inferences could be unlocked via our design where four source populations of each type are mixed together and then each of the two mixtures (one mixture per ecotype) are introduced into four restoration lakes. Some of these inferences would have been enhanced if we mixed the two ecotypes together (which we did in other lakes—see below), but our core ecological inference (benthic‐type versus limnetic‐type) meant that we could not mix these different ecotypes in most of our sites. Further, substantial trait variation among populations within a given ecotype (Haines et al., 2023) meant that the within‐ecotype mixed‐population design remained useful in the following respects.

1. *Replicate common garden experiments in nature*. Our mixture design means that four source populations of each type can be examined for their immediate responses to each of four different “common gardens” (i.e., all four populations of each type are placed into each of four different lakes). As such, we have a rare opportunity to quantify how trait variation is shaped by genetic effects (G—source population effect shared across lakes), environmental effects (E—restoration lake effect shared across source populations), and their interaction (GxE). Studies of this sort have been conducted in controlled settings, but we are not aware of any comparable studies for free‐living organisms in natural environments—at least for vertebrates.
2. *Local adaptation or the “baggage effect.”* By placing multiple source populations into each of multiple lakes, we also can test the determinants of population mean fitness—a long‐standing question in restoration ecology (Breed et al., 2013; Vergeer et al., 2008). At one extreme, the specific source population that is most successful following introduction might differ between the restoration lakes, perhaps because particular source populations have evolved in environments that are most similar to a particular restoration lake. In this scenario, local adaptation in the past has generated what amounts to the “pre‐adaptation” of particular source populations for particular restoration lakes—perhaps those that are most ecologically similar. At the other extreme, specific source populations could show the highest success across all restoration lakes (i.e., a “baggage effect” ‐ where populations bring their success with them to a new site), such as when particular source populations have the “best” genetic (e.g., low inbreeding or high heterozygosity) or environmental (e.g., low parasite load or high condition) backgrounds.
3. *Experiments in secondary contact*: By mixing the same source populations together in multiple natural environments, we are generating what amounts to replicate experiments in secondary contact between populations that evolved in isolation from each other. This mixture design thus affords opportunities to gain insight into several hypotheses about what happens when divergent populations are brought into secondary contact. At one extreme, mating could be most frequent between individuals from the same source population, such as in the case of co‐evolved sexual signals and preferences (Endler & Houde, 1995). At the other extreme, mating could be most frequent between individuals from *different* source populations, as can happen through a variety of mechanisms (Pfennig, 2007; Schwartz & Hendry, 2006). Furthermore, offspring from between‐population matings could show fitness that is higher than offspring from within‐population matings (as expected under inbreeding depression) or that is lower (as expected under outbreeding depression) (Ebert et al., 2002; Edmands, 1999; Fitzpatrick et al., 2020).
4. *Quantitative trait locus (QTL) mapping*: Even when a set of populations are selected to be similarly extreme along a particular axis of variation (here extreme benthic versus extreme limnetic), those populations will surely differ from each other in many of the genes contributing to that adaptation, as well as in evolutionary responses to other ecological axes (e.g., predation or parasitism). If those populations then interbreed, recombination will create individuals with diverse combinations of alleles across those genes, an appropriate scenario for the admixture mapping of QTL (Peichel & Marques, 2017). Our use of replicate mixtures of populations thus provides excellent potential for inferences about genotype–phenotype mapping. Crucially, each pool of introduced fish is replicated across multiple recipient lakes in each of which similar QTL crosses will be naturally generated. This replication of cross types across environments enables tests for the environmental dependence of QTL effects.

The design described above populates eight of the nine restoration lakes, with four receiving the benthic pool of fish and four receiving the limnetic pool of fish. What should be done with the remaining lake? In typical eco‐evolutionary experiments, some arenas are reserved for an absence of the focal species or for the introduction of related species (e.g., a congener). The idea behind such designs is to provide a treatment that allows one to compare the importance of intra‐specific variation to the importance of inter‐specific variation (Des Roches et al., 2018; Hendry, 2017). This type of treatment was not possible in our experiment because we were required by ADFG to restore all of the lakes with native fishes, which required the reintroduction of threespine stickleback in all cases. Thus, we instead decided on a new type of treatment for the final lake.

One important inference missing from the design thus far described centers on how benthic and limnetic individuals compare with each other—and interact—when placed together into a lake. We decided to facilitate this last set of inferences by releasing all eight source populations together into the final lake. With this last introduction, we can extend the above benefits of the “mixture” design to also (1) compare all eight populations together a single “common garden” (i.e., the same restoration lake), (2) assess how assortative mating and introgression between populations might be mediated by ecotype (a core question in studies of ecological speciation: McKinnon & Rundle, 2002; Nosil, 2012), and (3) conduct QTL admixture mapping studies with the full range of phenotypic variation across the eight populations of the two types. Of course, the use of only a single lake for this “all‐source” comparison would mean a lack of replication of this aspect of the experiment. However—as will be explained below—one of the benthic‐type introductions did not work, and so we later created an approximate “replicate” of this all‐source mixture in a second lake.

## 6 Q3. WHICH SPECIFIC SOURCE POPULATIONS SHOULD BE USED?

We needed to identify four benthic source populations and four limnetic source populations from our general study area—Cook Inlet, Alaska. This effort required sampling stickleback from a series of candidate lakes, quickly measuring those fish for traits that typically vary along the benthic/limnetic axis, and then identifying populations near the opposing ends of that axis. The candidate lakes (details in Appendix 1) were selected for sampling in three steps. First, a previous study used head shape measurements to place 45 populations from Cook Inlet along the benthic/limnetic morphology axis (Willacker et al., 2010). From that paper, we selected four of the five most benthic populations (Tern, Walby, Watson, and Corcoran) and the two most limnetic populations (Long and South Rolly). We also opportunistically sampled two intermediate populations from that paper (Finger and Echo). Second, based on the expectation that limnetic populations are typically found in large and deep oligotrophic lakes (Lavin & McPhail, 1986; Willacker et al., 2010), we sampled two such lakes on the Kenai Peninsula (Wik and Spirit). Third, as a backup, we sampled additional accessible lakes on the Kenai Peninsula (Arc, Ruth, Engineer, Jean). Finally, as a point of comparison, we sampled stickleback from the only restoration lake (G Lake) that still contained threespine stickleback before the rotenone treatment. That population was extirpated by the rotenone treatment later that year (2018).

**FIGURE 2:**
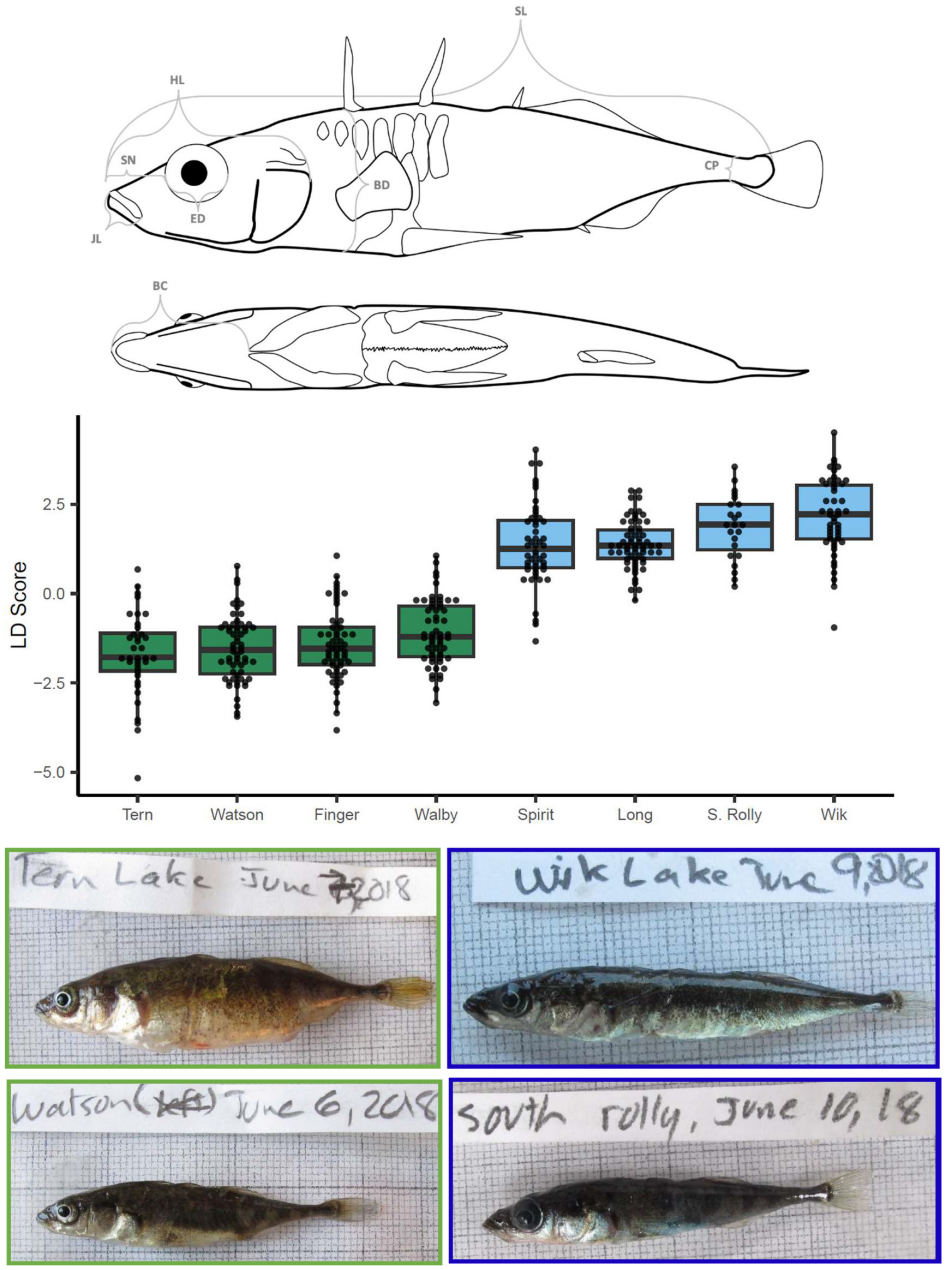
Summary of the morphological analyses used to polarize populations on the benthic‐to‐limnetic axis. The top panel shows some of the measured traits including standard length (SL), body depth (BD), buccal cavity length (BC), caudal peduncle width (CP), upper jaw length (JL), snout length (SN), eye diameter (ED), and head length (HL). The middle panel shows the results of the final linear discriminant analysis (LDA) that we used to confirm the four benthic and four limnetic source populations used for the experiment. The upper whisker extends from the hinge to the largest value no further than 1.5 times the IQR from the hinge (where IQR is the inter‐quartile range, or distance between the first and third quartiles). The lower whisker extends from the hinge to the smallest value at most 1.5 times the IQR of the hinge. Data beyond the end of the whiskers are called “outlying” points and are plotted individually. At the bottom are photographs of fish from the four most extreme source lake populations on the benthic/limnetic axis (see Figure 1). Note the relatively deeper bodies of fish from the benthic source lakes (at left, surrounded by green boxes).

From each candidate lake, we euthanized fish with an overdose of clove oil and then photographed them individually on 1 mm grid paper. The photographed fish were then preserved in 95% ethanol to also render them suitable for later genetic analysis. We next needed to rapidly (within 8 months) measure and statistically position each of the candidate populations on a benthic–limnetic axis that would decide which of the candidate populations should be chosen for our experiment. We did so by first measuring key trophic and body shape traits that previous work has shown to be associated with variation along the benthic–limnetic axis (Figure 2, Appendix 2).

After measuring those traits on 882 fish, we used linear discriminant analysis (LDA) to place each individual fish along the benthic–limnetic axis. This analysis used the *lda* function of the MASS R package, which uses the group sample sizes as prior probabilities to weight the covariance matrix used for the discriminant function. The analysis proceeded by first including fish from all sampled lakes with “lake” identity used for the discriminate classes. The two extremes of the resulting first LDA axis (Appendix 2) included lakes known (from Willacker et al., 2010) to have stickleback from the opposing benthic and limnetic ecotypes, confirming that the main axis of variation among lakes was indeed foraging morphology. Then, for various reasons (see Appendix 2 for details), we removed particular populations from further consideration and re‐ran the LDA with “ecotype” for the discriminant classes. Importantly, some candidate lakes were removed for scientific reasons (i.e., we wanted four genetically distinct populations of each ecotype), whereas other candidate lakes were excluded for practical reasons—echoing our earlier emphasis that “other considerations should also come into play.” For instance, one lake (Corcoran) was excluded because catch rates were too low. Furthermore, all source lakes required reliable access points.

**FIGURE 3:**
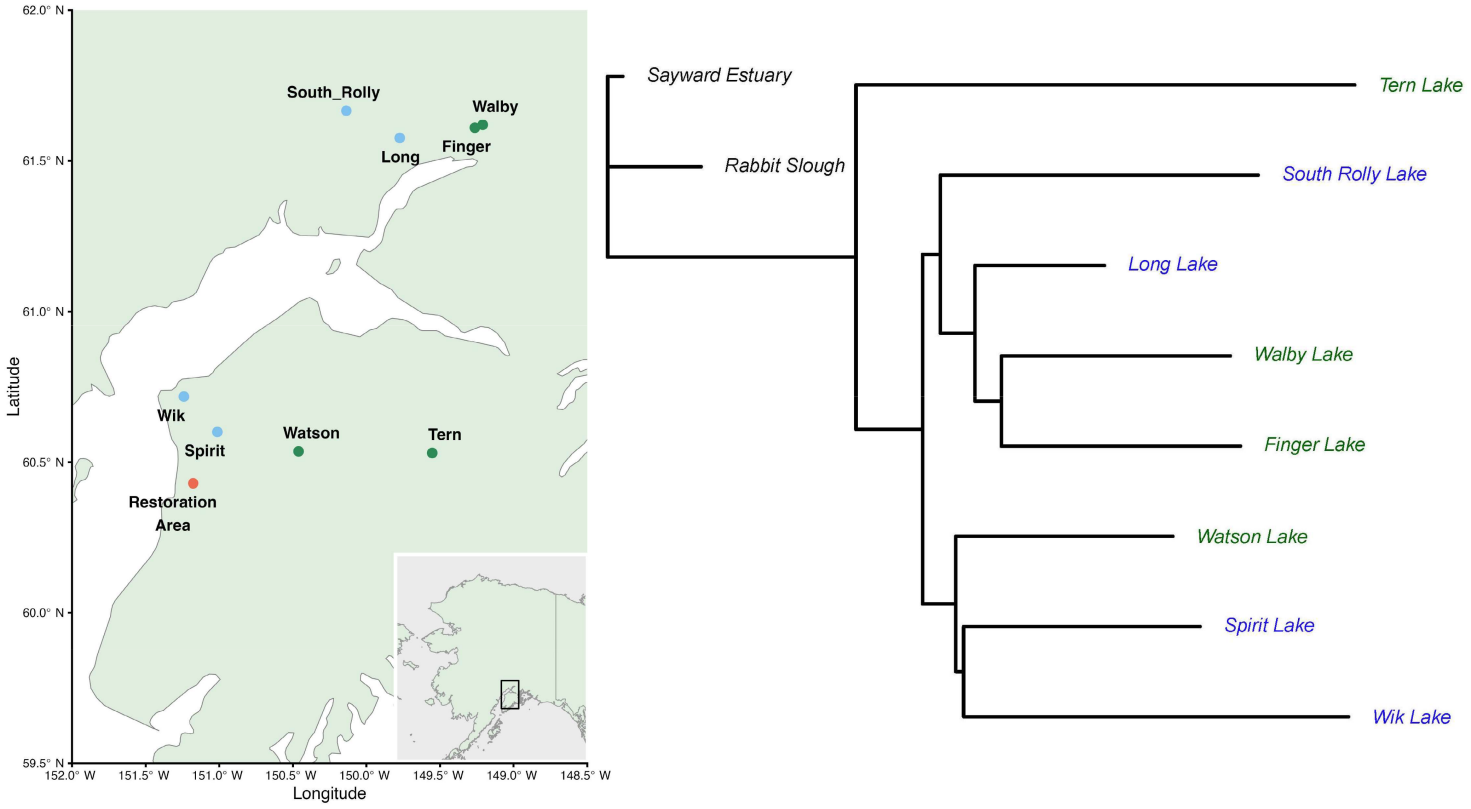
At left is a map of the location of the restoration lakes (one point for the restoration area) and the source lakes (one point per lake, with those containing benthic ecotypes in green and those containing limnetic ecotypes in blue). At right is a depiction of genetic relationships among the source lake populations and two anadromous populations (Sayward Estuary and Rabbit Slough), based on whole‐genome allele frequency data. For this genomic analysis, we pooled extracted DNA from 100 individuals per population in equimolar amounts and sequenced to a target mean depth of 200× to obtain allele frequencies. We calculated mean *F*_ST_ (for all SNPs on Chromosome 1) between each pairwise combination of populations (see also Table 1) and built the tree using a distance‐based neighbor‐joining algorithm. Branch lengths are determined by F_ST_ between populations.

The final LDA (Figure 2), which  included the "best" eight candidate lakes (four of each ecotype), successfully assigned 94.5% of the individual fish to the correct ecotype. These final lakes selected to be sources for the experiment included two benthic populations (Finger and Walby) and two limnetic populations (Long and South Rolly) from the Mat‐Su area; and two benthic populations (Tern and Watson) and two limnetic populations (Spirit and Wik) from the Kenai Peninsula (Figure 3). As expected from previous work, the lakes with benthic ecotypes were shallower and more productive than the lakes containing limnetic ecotypes (Tables 1 and 2; Appendix 3). Further, two populations of each ecotype came from each of two different geographic areas (Mat‐Su vs. Kenai), thus potentially enabling inferences about regional effects independent of (or interacting with) ecotype. Additional analyses of these samples following their selection for the introductions are reported in Haines et al. (2023).

**TABLE 1:** Locations and basic properties of the source lakes (above dashed line) and restoration lakes (below dashed line).

Lake	Region	Latitude	Longitude	Ecotype	Surface area (ha)	Max depth (m)
Tern	Kenai	60°31′49.7″	149°33′12.6″	B	44.1	2.9
Watson	Kenai	60°32′09.1″	150°27′42.7″	B	22.8	4.3
Finger	Mat‐Su	61°36′34.3″	149°15′52.2″	B	135.7	13.4
Walby	Mat‐Su	61°04′23.3″	149°46′18.1″	B	19.6	5.5
Spirit	Kenai	60°36′01.1″	151°00′45.2″	L	123.2	21.0
Long	Mat‐Su	61°34′32.1″	149°46′25.4″	L	16.9	7.9
South Rolly	Mat‐Su	61°40′00.3″	150°08′12.7″	L	43.8	19.2
Wik	Kenai	60°43′02.8″	151°14′30.3″	L	74.1	24.4
CC	Kenai	60°25′18.4″	151°11′41.5″	B	1.8	3.1
Leisure L.	Kenai	60°24′52.9″	151°12′39.0″	B	4.5	7.1
Leisure P.	Kenai	60°25′09.7″	151°12′24.7″	B	0.6	3.1
Crystal	Kenai	60°25′27.1″	151°11′38.8″	L	6.8	10.0
Fred's	Kenai	60°25′23.3″	151°11′54.7″	L	2.5	1.2
Hope	Kenai	60°25′17.5″	151°11′17.8″	L	10.9	8.9
Ranchero	Kenai	60°25′22.3″	151°10′58.0″	L	3.1	3.1
G	Kenai	60°25′47.6″	151°10′37.7″	B & L	7.0	9.4
Loon	Kenai	60°31′13.7″	151°03′06.6″	B & L	8.9	6.5

*Note*: In the source lakes, “ecotype” represents the stickleback ecotype (B = benthic; L = limnetic) that evolved and is resident in that lake. In the restoration lakes, “ecotype” refers to the stickleback ecotype that was introduced to that lake in this study. Note that only benthic fish were introduced into G Lake in 2019 but that introduction was unsuccessful, and so both types were introduced in 2022.

**TABLE 2:** Environmental characteristics of source lakes (above the dashed line) and restoration lakes (below the dashed line)—as mean values across all measurements.

Lake	TP (μg/L)	TN (mg/L)	DOC (mg/L)	pH	Sp Cond (μS/cm)	Ca (mg/L)	Chl‐*a* (μg/L)	Z_secchi_ (m)
Tern	8.4	0.595	1.1	8.1	142.7	29.7	0.6	Bottom
Watson	14.1	0.260	5.3	7.9	98.6	19.2	0.8	3.0
Finger	12.7	0.413	5.6	8.4	239.0	27.7	3.3	3.0
Walby	8.4	0.402	6.3	8.7	166.7	27.3	1.8	4.3
Spirit	5.5	0.196	7.7	7.9	61.8	9.2	0.6	9.2
Long	5.4	0.264	4.5	8.1	78.2	13.3	0.9	5.1
South Rolly	4.7	0.223	8.0	7.5	31.7	5.6	0.9	3.9
Wik	2.7	0.181	2.7	7.6	14.9	1.3	0.4	9.35
CC	8.3	0.272	9.6	7.2	48.0	3.9	1.1	2.6
Leisure L.	8.9	0.298	11.8	7.6	42.8	4.4	1.6	2.6
Leisure P.	13.3	0.243	10.3	7.1	48.5	5.5	1.3	1.7
Crystal	7.5	0.265	5.2	7.7	67.2	6.0	0.9	5.3
Fred's	7.5	0.386	6.8	8.4	60.1	4.8	0.9	Bottom
Hope	8.2	0.253	4.7	7.6	70.9	7.3	1.2	3.6
Ranchero	13.9	0.360	5.5	7.5	60.3	5.9	2.6	2.4
G	4.7	0.205	4.2	6.8	11.3	0.6	0.4	6.0
Loon	9.6	0.402	9.5	6.9	18.1	1.3	1.3	3.3

*Note*: Variables include total phosphorus (TP, μg/L), total nitrogen (TN, μg/L), dissolved organic carbon (DOC, mg/L), pH, specific conductance (SpCond, μS/cm at 25°C), calcium (Ca, mg/L), chlorophyll *a* (Chl*a*, μg/L) as an estimate of phytoplankton biomass, and Secchi depth (Z_secch_i, m) as an estimate of transparency. “Bottom” indicates that the Secchi disk could be seen all the way to the bottom of these shallow lakes. Water chemistry was collected in early June of 2018 and 2019, just prior to stickleback additions in restoration lakes in 2019, and averaged between years for lakes in which two reliable values were available. For Ca, data were only collected in 2018.

For optimal inferences in our experiment, the benthic and limnetic ecotypes would not have a single evolutionary origin and the different source populations would be genetically distinct from each other. We confirmed these properties based on whole‐genome pool‐sequencing on an Illumina Hi‐Seq, with pools of 100 individuals per population. The bioinformatics steps are described in Weber et al. (2022). We here report F_ST_ values between each pair of populations averaged across all SNPs on Chromosome 1 (for computational speed). The resulting table of F_ST_ values between populations (Table 3) was converted into an estimate of the phylogenetic tree using neighbor joining implemented in the ape package in R, rooting the tree with the anadromous population from Sayward Estuary, Vancouver Island (Figure 3). We also included a central Cook Inlet anadromous population (Rabbit Slough, Alaska), which clustered with the Sayward population. The deepest split among the source populations corresponded to region (Mat‐Su vs. Kenai), and the benthic and limnetic populations did not form separate monophyletic groups, suggesting that the ecotypes evolved multiple independent times. Furthermore, all of the source populations were genetically distinct from each other (all pairwise *F*_ST_ > 0.17), indicating minimal contemporary gene flow even at very small geographic distances (2 km between Walby and Finger, yet *F*_ST_ = 0.20). As expected, the two anadromous populations were genetically similar (*F*_ST_ = 0.05) despite the large distance between them (>2000 km), and they were similarly divergent from each of the source populations (*F*_ST_ > 0.25).

**TABLE 3:** *F*_ST_ estimates between each pair of source populations and two marine populations (Sayward Estuary on Vancouver Island, and Rabbit Slough in the Mat‐Su Valley of Alaska—shaded columns) for all SNPs on Chromosome 1.

	Finger Lake	Sayward Estuary	Long Lake	Rabbit Slough	South Rolly Lake	Spirit Lake	Tern Lake	Walby Lake	Watson Lake	Wik Lake
Finger Lake		0.27	0.17	0.30	0.26	0.27	0.37	0.20	0.23	0.31
Sayward Estuary	0.27		0.22	0.05	0.27	0.26	0.33	0.26	0.25	0.31
Long Lake	0.17	0.22		0.26	0.20	0.20	0.32	0.16	0.18	0.26
Rabbit Slough	0.30	0.05	0.26		0.30	0.30	0.35	0.29	0.29	0.34
South Rolly Lake	0.26	0.27	0.20	0.30		0.26	0.40	0.27	0.25	0.32
Spirit Lake	0.27	0.26	0.20	0.30	0.26		0.35	0.25	0.19	0.26
Tern Lake	0.37	0.33	0.32	0.35	0.40	0.35		0.36	0.34	0.43
Walby Lake	0.20	0.26	0.16	0.29	0.27	0.25	0.36		0.22	0.32
Watson Lake	0.23	0.25	0.18	0.29	0.25	0.19	0.34	0.22		0.26
Wik Lake	0.31	0.31	0.26	0.34	0.32	0.26	0.43	0.32	0.26	

## 7 Q4. WHICH ECOTYPE SHOULD GO INTO WHICH RESTORATION LAKE?

**FIGURE 4:**
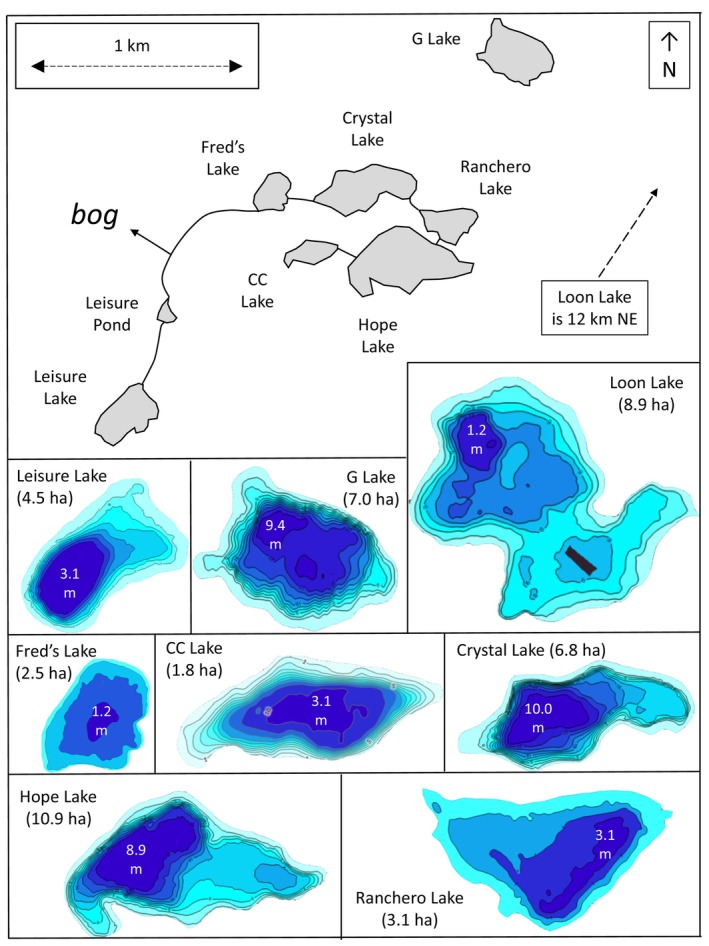
Visual summary of the restoration lakes. The top panel shows the geographical layout and drainage connections of all restoration lakes except Loon Lake, which is 12 km to the NE. The bottom panel shows depth profiles (2017 DigitalGlobe; Microsoft Corporation) for all lakes except Leisure Pond, which is too small for such data. Note that scales, including the depth profiles, differ among the panels. The black rectangle on Loon Lake is an artefact of the data source.

Another important decision was which restoration lakes should receive which ecotype. Compared with the large environmental differences between the source lakes that contained benthic stickleback and the source lakes that contained limnetic stickleback, the restoration lakes were relatively similar to each other (Tables 1 and 2; Appendix 3). Overall, the restoration lakes were some what similar in depth and water clarity to the benthic source lakes, but they had more dissolved organic carbon than all of the source lakes (Tables 1 and 2; Appendix 3). As such, environmental variables were not our first consideration as to which ecotypes should be placed into which restoration lake. Instead, our primary consideration became watershed connections between the restoration lakes (Figure 4)—because connectivity could lead to mixing of the fish introduced into different lakes. Hence, we reasoned that lakes with the closest connections should receive the same ecotype. As a result, we decided to introduce the limnetic ecotype into the four lakes with the most obvious watershed connections: Hope Lake flows into Ranchero Lake which flows into Crystal Lake which flows into Fred's Lake (Although CC Lake flows into Hope Lake, the flow is extremely low and ephemeral.)

The eight restoration lakes varied so much in size (Table 1) that introducing the same *number* of fish into each lake would mean a huge discrepancy in initial stocking density between the restoration lakes, a situation that was not acceptable to ADFG. Hence, we needed to somehow scale the number of introduced fish by the size of the restoration lake—but this scaling could not be linear. For instance, if 400 fish (100 from each source lake) was the minimum appropriate number to introduce into the smallest lake (Leisure Pond), linear scaling by lake surface area would require that a logistically infeasible number of 7267 fish be introduced into Hope Lake. By contrast if 2000 fish was considered a logistically feasible number for introduction to Hope Lake, then linear scaling by surface area would mean an unreasonably low number of 111 total fish for introduction into Leisure Pond. Thus, in consultation with ADFG, we settled on non‐linear scaling from 400 total fish (the minimum number that we considered reasonable from a genetic perspective) into the each of two smallest lakes (Fred's and Leisure Pond), 800 total fish into each of the next two largest lakes (CC and Ranchero), and 1600 total fish into each of the remaining four larger lakes. Finally, for Loon Lake, which received all eight source populations, we planned for 2000 total fish representing 250 from each source lake.

## 8 IMPLEMENTATION—THE INITIAL INTRODUCTIONS

The boxer Mike Tyson once said “everyone has a plan until they get punched in the mouth,” echoing a similar older sentiment that “no plan survives first contact with the enemy.” Thus, even armed with what we considered to be the best possible plan, we fully anticipated encountering problems that required on‐the‐fly modifications, which proved to be the case. We outline these circumstances in more detail in Appendix 4, whereas we here summarize some of the problems that emerged and are instructive regarding how to design and implement experiments in real‐world situations.

Implementing our experiment required a very complicated, intricate, and labor‐intensive process. First, more than 10,000 fish had to be captured in a series of phases across eight source lakes that were separated by up to 5 h of driving (and more depending on traffic). The fish had to be transported to a field laboratory alive and maintained in good health in source‐population specific bins. Each fish then had to be individually photographed and fin‐clipped and placed into specific mixtures for introduction into the different restoration lakes. To avoid holding fish for too long during this processing, the releases had to take place in a series of waves of equal abundance into the different restoration lakes at the same time. Despite a crew of more than 10 people, implementing the experiment took almost 1 month. The approach of introducing fish in waves helped to standardize the number of fish introduced into the different lakes at any given time, but it also meant that introductions into the larger lakes (which received more fish) continued for several weeks after introductions into the smaller lakes (which received fewer fish) had ceased.

The experiment could only start in late May of 2019 when all of the lakes were sure to have lost their ice, and when the rotenone that was applied in the fall of 2018 had "cleared." Yet the air temperatures by mid‐June had reached levels that threatened stickleback mortality during processing. We reduced this problem by continually adding ice packs to the bins holding fish, yet mortality increased as time went on. Fish that died before being placed into the mixtures could be removed and replaced with new fish from the same source, whereas fish that died after mixing could be removed but their source population was not immediately known. As a result, some unknown deviations from our planned mixtures will have occurred. The fish that died after mixing were preserved and will have to be genotyped to confirm their population of origin before we can know the precise numbers of fish from each source lake that were introduced into each restoration lake.

We had worried (see above) that particular source populations might be especially susceptible to transport and handling stress. Fortunately, fish from seven of the eight sources seemed to have no trouble in this respect—because their mortality was very low (apart from the above increase when temperatures got too high). Fish from Tern Lake, however, were problematic in that they proved hard to catch at certain times (but not at other times), and they also were more prone to pre‐processing mortality. Notably, numerous naturally dead fish were found along the shoreline of Tern Lake when we were not sampling, indicating an extensive natural die‐off occurred (We have some hypotheses for higher mortality of Tern Lake fish, but they are too speculative to detail here.) Eventually, it became clear that it would not be feasible, responsible, or practical to keep ramping up sampling from Tern Lake in hopes of matching numbers from the other source lakes. Hence, we decided to under‐represent Tern relative to the other benthic lakes in some of the larger restoration lakes (G, Leisure Lake, and Loon). We matched this imbalance in the benthic fish introductions by similarly under‐representing the numbers of fish from one limnetic source lake (Wik—owing to accessibility complications) introduced into the three largest lakes to receive that ecotype (Hope, Crystal, and Loon).

The above departure from introducing a balanced mixture of source populations into each restoration lake (Table 4) was initially irksome and led to an apologetic tone in our discussions of the aspirational design versus the final implementation. However—as a reviewer of this manuscript helped us to understand—balanced contributions to introduced mixtures are not only unnecessary, they also are not necessarily optimal. Take for instance a situation where one habitat type is much more abundant than another. It such cases, it might be beneficial to mostly introduce genotypes best suited for that habitat, while also increasing diversity and evolutionary options by introducing smaller numbers of genotypes expected to be better suited to less‐common habitats. Furthermore, any general fragility of particular genotypes (e.g., Tern Lake fish in our case) would suggest that their under‐representation in restoration efforts would generally improve restoration success.

## 9 SUCCESS, FAILURE, AND SUCCESS ANEW

Immediately prior to our introductions in 2019, no stickleback could be captured in any of the post‐rotonone restoration lakes—a result in striking contrast to the ease of capturing stickleback in nearby lakes (Massengill, 2022). This result conforms with expectations that (1) rotenone is an effective agent at eliminating fish from lakes, and (2) the restoration lakes were not connected to other lakes that could serve for natural colonization. By striking contrast, we were able to easily collect numerous stickleback from the restoration lakes (with one exception—see below) in 2020 and in all subsequent years. Thus, although we cannot be entirely sure that all stickleback subsequently caught in these lakes are derived from our initial introductions, that simple expectation does seem likely. Indeed, genetic analysis of fish captured from these lakes has unambiguously assigned each fish to one or more of the specific source populations (L. Eckert, unpublished data).

In striking contrast with the other eight lakes, no stickleback could be captured in G Lake by ADFG or by our group in 2020 or 2021, despite the fact that it was easy to capture stickleback in that very lake prior to the rotenone treatment (Andrew P. Hendry and Robert L. Massengill unpublished data). (Remember, it was the only restoration lake to have resident stickleback at the time of the rotonone treatment.) We therefore inferred that our initial introduction into G Lake had failed to produce a viable population. Several ideas have been advanced for the failure of this introduction, including residual rotenone (unlikely—as per Couture et al., 2022), low prey availability (zooplankton and benthic macroinvertebrates were low after rotenone, unpublished data), particular water chemistry (very low Ca + levels, Table 2), high predation (many stocked salmonids could be seen in the littoral area: pers. obs.), or an environment/phenotype mismatch (G Lake was the most limnetic‐like lake and yet it received benthic fish only). We cannot distinguish between these possibilities, but several of these problems were expected to lessen with time. Indeed, continued sampling showed recovering zooplankton and benthic invertebrate communities (Alison M. Derry unpublished data). We therefore decided to attempt another introduction into G Lake in 2022.

We made several changes to our procedures for this reintroduction in hopes of maximizing the chances of success on this second effort in G Lake. First, we decided to reintroduce stickleback in higher numbers than before in hopes of providing the population a demographic boost at the outset. Second, we decided to introduce all eight source populations in hopes of reducing the striking environment/phenotype mismatch from our first introduction. Furthermore, the benefits of replicating an “all sources” mixture appeared greater than attempting again a fourth replicate for the benthic‐only ecotype treatment—especially given the previous failure of that type to become established in that lake.

The reintroduction was implemented May 16–25, 2022, following the same procedures as the 2019 introductions described above—with a few exceptions. First, only a few fish were captured in one original source lake (Long Lake), where pike had recently become numerous and seemingly caused a dramatic decrease in the local stickleback population. As a result, we had to drop Long Lake from the reintroduction, leaving us with four benthic sources and three limnetic sources for the reintroduction into G Lake. Second, the fish were kept in source‐lake specific bins after processing but before introduction, which allowed us to replace any mortalities before release with the same number of fish from the same source lake. A total of 3495 fish were released, 495 from Wik Lake and 500 from each of the other six source lakes. Sampling in 2023 revealed that this second introduction to G Lake was successful—with many fish captured along the entire shoreline of the lake. As a result, the final experimental design included four lakes receiving limnetic fish, three lakes receiving benthic fish, and two lakes receiving both ecotypes (Figure 1; Table 4).

**TABLE 4:** Numbers of stickleback from each source lake (columns) introduced into each restoration lake (rows).

Restoration lakes	“Limnetic” Source Lakes				“Benthic” Source Lakes			
	Long	South Rolly	Spirit	Wik	Finger	Tern	Watson	Walby
G (2019)	0	0	0	0	*445*	*302*	*452*	*449*
Leisure L.	0	0	0	0	400	278	406	419
CC	0	0	0	0	202	191	202	202
Leisure P.	0	0	0	0	103	102	105	102
Hope	455	444	461	294	0	0	0	0
Crystal	400	400	400	294	0	0	0	0
Ranchero	203	203	202	198	0	0	0	0
Fred's	104	109	103	103	0	0	0	0
Loon	287	275	300	172	299	170	294	301
G (2022)	0	500	500	500	500	495	500	500

*Note*: The restoration lakes above the dashed line received fish from four source lakes of a given ecotype (benthic or limnetic), whereas the restoration lakes below the dashed line received fish from all eight source lakes (Loon) or seven of the eight source lakes (G). Note that G Lake appears twice in the table because the first introduction of benthic source fish in 2019 (top row) did not work (numbers in italics in the top row), whereas the second introduction of seven of the eight source lakes in 2022 (bottom row) did work. Although the total number of fish introduced into a given restoration lake is accurately reflected in this table, the numbers per source lake in a given restoration lake are approximate—owing to some mortality between the creation of mixtures and their release into the restoration lakes (see Appendix 4).

## 10 CONCLUSIONS

Eco‐evolutionary experiments in a restoration setting are certainly encumbered by some important constraints—such as limited replication (we had “only” nine lakes to work with), various exigencies (in our case, high temperature toward the end of the experiment), and massive effort over a constrained period (in our experiment, more than 10,000 fish were individually photographed and fin‐clipped in less than a month). Furthermore, optimal design from a conceptual or inferential standpoint might not be fully compatible with the needs of management agencies or other stakeholders. In our case, we were not able to establish control lakes in which the focal species was not introduced. Furthermore, management agencies had to conduct other manipulations (stocking salmonid fishes—as described in Massengill, 2022) to appease landowners concerned with ecosystem services lost when their fishable invasive species was extirpated. Any of these manipulations could confound or modify or degrade the emergent signal of our experimental treatment (the introduction of benthic versus limnetic stickleback). Yet, when adding eco‐evolutionary experiments to restoration efforts, it is important to not compromise restoration goals for the sake of scientific insight. Indeed, many of our decisions sought to optimize this balance—as our experiments would not work if restoration failed. Further, if restoration failed, it would undermine our argument for adding experiments to restoration efforts. Fortunately, experiments designed to dove‐tail well with restoration goals might serve to improve restoration success at the same time as generating novel and general scientific insight.

Despite potential practical constraints, eco‐evolutionary experiments conducted in restoration settings offer several key opportunities and advantages (see also LaRue et al., 2017). Perhaps most obviously, restoration might be the only context where multiple locations in the real world *require* the introduction of focal species. Furthermore, restoration efforts often have built‐in monitoring programs that can be modified to collect data suitable for eco‐evolutionary inference. In our case, data collected at regular intervals by ADFG can be integrated with our continued annual monitoring of the stickleback populations (phenotypes and genotypes) and the restoration lakes (limnological parameters and food webs). Furthermore, the oft‐stated limitation of conducting experiments in nature could be considered a profound strength of such efforts. That is, the restoration context includes—as would any other experiment in the “real world”—uncontrolled variation among ostensible “replicates.” We consider this last fact to be a benefit rather than a constraint—because natural variation among locations is always an integral feature of the real world. Understanding the importance of a given evolutionary treatment (e.g., two types of a species) therefore requires assessing how those effects vary across a real range of uncontrolled variation in other factors.

Overall, we hope that this paper contextualizes the experiments we conducted, and also provides advice—perhaps even inspiration—for researchers considering whether to conduct eco‐evolutionary experiments in natural settings. Eco‐evolutionary experiments in nature are not impossible, nor even impractical; they are just very difficult. We anticipate that the difficulty of such work will more than pay‐off through much‐needed insights into the importance of intraspecific diversity and contemporary evolution in “real” contexts—as opposed to artificially controlled venues.

## AUTHOR CONTRIBUTIONS

**Andrew P. Hendry:** Conceptualization (lead); funding acquisition (equal); investigation (equal); methodology (equal); project administration (lead); resources (equal); supervision (equal); visualization (equal); writing – original draft (lead); writing – review and editing (lead). **Rowan D. H. Barrett:** Conceptualization (equal); funding acquisition (equal); investigation (equal); methodology (equal); project administration (supporting); resources (equal); supervision (equal); writing – review and editing (supporting). **Alison M. Bell:** Conceptualization (supporting); funding acquisition (supporting); investigation (equal); methodology (supporting); project administration (supporting); resources (equal); supervision (equal); writing – review and editing (supporting). **Michael A. Bell:** Conceptualization (supporting). **Daniel I. Bolnick:** Conceptualization (equal); formal analysis (equal); funding acquisition (equal); investigation (equal); methodology (equal); project administration (supporting); resources (equal); supervision (equal); visualization (equal); writing – review and editing (supporting). **Kiyoko M. Gotanda:** Conceptualization (supporting); funding acquisition (supporting); investigation (equal); resources (supporting); supervision (equal); writing – review and editing (supporting). **Grant E. Haines:** Conceptualization (supporting); data curation (equal); formal analysis (equal); investigation (equal); methodology (supporting); visualization (equal); writing – original draft (supporting); writing – review and editing (supporting). **Åsa J. Lind:** Data curation (equal); investigation (supporting); methodology (supporting); project administration (supporting); writing – review and editing (supporting). **Michelle Packer:** Data curation (equal); methodology (supporting); project administration (equal). **Catherine L. Peichel:** Conceptualization (equal); funding acquisition (equal); investigation (equal); methodology (equal); project administration (supporting); resources (equal); supervision (equal); writing – review and editing (supporting). **Christopher R. Peterson:** Investigation (supporting). **Hilary A. Poore:** Data curation (equal); investigation (equal); methodology (equal); project administration (equal); supervision (equal). **Robert L. Massengill:** Conceptualization (supporting); funding acquisition (supporting); investigation (supporting); project administration (supporting); resources (supporting); writing – review and editing (supporting). **Kathryn Milligan‐McClellan:** Conceptualization (equal); funding acquisition (equal); investigation (equal); methodology (equal); project administration (supporting); resources (equal); supervision (equal); writing – review and editing (supporting). **Natalie C. Steinel:** Conceptualization (equal); funding acquisition (equal); investigation (equal); methodology (equal); project administration (supporting); resources (equal); supervision (equal); writing – review and editing (supporting). **Sarah Sanderson:** Investigation (supporting). **Matthew R. Walsh:** Conceptualization (equal); funding acquisition (equal); investigation (equal); methodology (equal); project administration (supporting); resources (equal); supervision (equal); writing – review and editing (supporting). **Jesse N. Weber:** Conceptualization (equal); funding acquisition (equal); investigation (equal); methodology (equal); project administration (supporting); resources (equal); supervision (equal); writing – review and editing (supporting). **Alison M. Derry:** Conceptualization (lead); data curation (equal); formal analysis (equal); funding acquisition (equal); investigation (equal); methodology (equal); project administration (equal); resources (equal); supervision (equal); writing – review and editing (supporting).

## FUNDING INFORMATION

The study received funding from *Hendry*: Canada Research Chair Tier 1 & NSERC Discovery Grant (RGPIN‐2018‐04761), *Barrett*: Canada Research Chair Tier 2 & NSERC Discovery Grant (RGPIN‐2019‐04549), *M. Bell*: NIH (1R01GM124330–01), *Bolnick*: US NSF, Directorate for Biological Sciences (DMS‐17168‐03), *Milligan‐McClellan*: NIH (R15GM122037), *Peichel*: Swiss National Science Foundation (TMAG‐3‐209309/1), *Weber*: NIH (1R35GM142891–01), and *Derry*: NSERC Discovery Grant (RGPIN‐2022‐03706).

## CONFLICT OF INTEREST STATEMENT

The authors have no conflict of interest to declare.

## Figures and Tables

**Figure:**
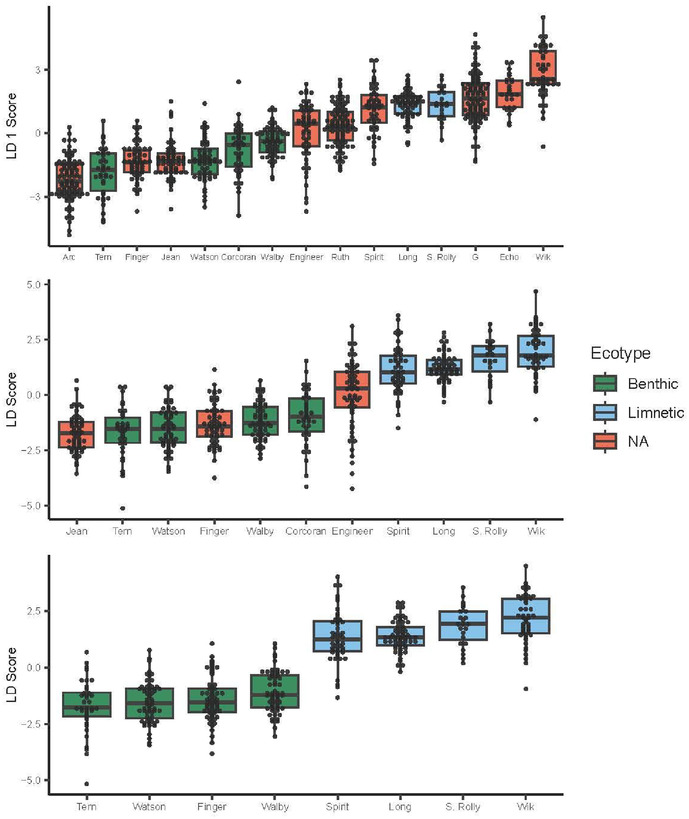
The top panel shows results of the first linear discriminant analysis (LDA) that included all of the candidate populations, arranged on the first axis discriminating among lakes. Individual data points correspond to locations of each individual fish along the axis, the boxes include 50% of the fish in each population, and the whiskers contain 95% of the fish in each population. Ecotype designations in this panel correspond to designations in the Willacker et al. (2010) paper, and here “NA” indicates populations not included in that paper. The middle panel removes some populations (see explanations above) and now assigns populations to the benthic and limnetic categories (“NA” here indicates populations subsequently removed to generate the bottom panel). The bottom panel shows the final results for how individual fish and population averages fall on the benthic‐to‐limnetic axis—and it is the same as that shown in Figure 2 of the main text.

**Figure:**
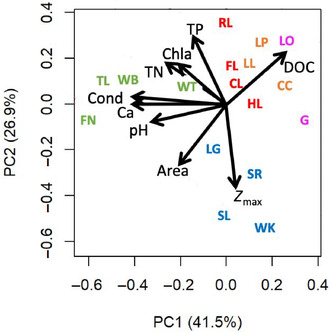
Principal component analysis of the environmental variables across the source and restoration lakes. Blue labels indicate source lakes containing limnetic stickleback ecotypes: Long (LG), Spirit (SL), South Rolley (SR), and Wik (WK). Green labels indicate source lakes containing benthic stickleback ecotypes: Finger (FN), Tern (TL), Walby (WB), and Watson (WT). Red labels indicate restoration lakes that received limnetic stickleback ecotypes: Crystal Lake (CL), Fred's Lake (FL), Hope Lake (HL), and Ranchero Lake (RL). Orange labels indicate restoration lakes that received benthic stickleback ecotypes: CC Lake (CC), Leisure Lake (LL), and Leisure Pond (LP). Pink labels indicate restoration lakes that received both limnetic and benthic stickleback ecotypes: G Lake (G) and Loon Lake (LO). Abbreviations for environmental variables are as follows: calcium (Ca), chlorophyll *a* (chl‐a), dissolved organic carbon (DOC), lake surface area (Area), maximum depth (Zmax), pH, specific conductance (Cond), total nitrogen (TN), and total phosphorus (TP).

## Data Availability

Phenotype data, environmental data (including the coordinates used to make the map), and R code for the LDAs and figures presented in this article are available on github at github.com/ghaines3/AK‐stickleback‐intro‐selection.

## APPENDIX 1 LAKES CONSIDERED AS CANDIDATE SOURCES FOR THE EXPERIMENT

The table provides the locations of the candidate lakes, and the number of fish from each used for the linear discriminant analyses (LDA) categorizing stickleback from the lakes into the benthic and limnetic ecotypes. This table shows all lakes included in the first LDA, with lakes then removed sequentially and the LDA modified as described in Appendix 2.

**Table:**

Lake	Region	Latitude (N)	Longitude (W)	Number of fish analyzed
Arc	Kenai	60°26′58.9″	151°06′15.8″	96
Corcoran	Mat‐Su	61°34′24.1″	149°41′31.3″	41
Echo	Kenai	60°26′18.2″	151°09′27.7″	27
Engineer	Kenai	60°28′34.3″	150°19′34.9″	60
Finger	Mat‐Su	61°36′34.3	149°15′52.2	59
G	Kenai	60°25′47.6	151°10′37.7	100
Jean	Kenai	60°30′13.2″	150°10′04.3″	60
Long	Mat‐Su	61°34′32.1	149°46′25.4	62
Ruth	Kenai	60°24′53.0″	151°09′54.6″	99
South Rolly	Mat‐Su	61°40′00.3	150°08′12.7	23
Spirit	Kenai	60°36′01.1	151°00′45.2	50
Tern	Kenai	60°31′49.7	149°33′12.6	37
Walby	Mat‐Su	61°04′23.3	149°46′18.1	59
Watson	Kenai	60°32′09.1	150°27′42.7	60
Wik	Kenai	60°43′02.8	151°14′30.3	49

## APPENDIX 2 MORPHOLOGICAL MEASUREMENTS AND LINEAR DISCRIMINANT FUNCTIONS

Photographs were taken with a Nikon D800 (Finger, Walby, and 31 Long Lake fish) or a Canon PowerShot G11 (all other fish). Two cameras were necessary because one camera failed during the fieldwork and had to be replaced. The measured traits (see Figure 2 in the main body of the paper) were body mass, standard length (SL), body depth (BD), buccal cavity length (BC), caudal peduncle width (CP), upper jaw length (JL), snout length (SN), eye diameter (ED), and head length (HL). Body mass was measured on a digital scale (to 0.01 g) and SL, BD, and BC were measured with digital calipers (to 0.01 mm). The other traits were measured from the photographs in ImageJ.

As most traits covary with body size, we needed to standardize them to a common body size. The optimal approach here is to use the allometric equation *M*_s_ *= M*_obs_(*L*_s_/*L*_obs_)^*b*^, where *M*_obs_ is the measured trait value, *L*_s_ is the mean length, *L*_obs_ is the observed length, and *b* is the common within‐group slope (i.e., not considering population interactions) calculated from a linear model of the form log(trait) ~ log(SL) + Lake. For the initial analysis on which decisions were based, a coding error meant that we did not use the correct equation for size standardization, because the trait measurement and standard length had not been log transformed. However, we later corrected this error, and it did not change inferences about the four most appropriate populations of each type. Hence, we here present results based on the correct version.

The first LDA (top panel in the figure below) was trained to assign each fish to one of the 15 sampled populations, thus identifying the major axis of differentiation among lakes. The first axis of this LDA explained 42.8% of the variance of all LD axes, and it had an assignment accuracy rate to “home” lakes of 63.9%. Examination of trait loadings confirmed that LD1 was strongly associated with a benthic–limnetic axis of variation, and it was also similar to the benthic–limnetic axis found by Willacker et al. (2010) for a subset of the same populations. After this first analysis, four lakes were removed from consideration. G Lake was removed because the stickleback population in contained was extirpated by the rotenone treatment. Arc Lake was removed because it was treated with rotenone in 2008, and it appears to have been recently derived from marine ancestors—as all of the individuals were fully plated. Ruth Lake was removed because it was morphologically intermediate along the benthic/limnetic axis. Echo Lake was removed because of low catch rates and access issues.

For the next LDA (middle panel in the figure below), we assigned fish from the remaining 11 lakes to benthic versus limnetic ecotypes. This new ecotype LDA was trained by referring to the results of the previous analysis (both Willacker et al., 2010, and the first LDA described above) to designate Tern, Watson, Walby, and Corcoran as benthic populations, and Wik, Long, South Rolly, and Spirit as limnetic populations. This new analysis correctly assigned individual fish from these lakes to their respective ecotype at a rate of 92.1%. Based on this new analysis, we next removed the Engineer from further consideration due to their intermediate morphology. We also removed Jean due to (1) a strong mismatch between the morphology we measured (benthic‐like) and the morphology expected based on physical characteristics of the lake (it is 20 m deep lake for which limnetic fish would be expected), (2) a recent change in armor morphology (comparing our measurements to those reported in Bell & Ortí, 1994), and (3) balanced geographical representation (we wanted two benthic and two limnetic populations from each of the Mat‐Su area and the Kenai Peninsula). Furthermore, we replaced Corcoran with Finger in the benthic category because the latter appeared more benthic and because catch rates in Corcoran Lake were too low for its use in the experiment. The final LDA (bottom panel in the figure below) based on the remaining eight lakes (four of each ecotype) successfully assigned 94.5% of the individual fish to the correct ecotype.

## APPENDIX 3 DETAILED METHODS USED TO DETERMINE ENVIRONMENTAL CONDITIONS IN THE SOURCE AND RESTORATION LAKES

Dissolved oxygen (DO; mg/L), pH, water temperature (°C), and conductivity (μS/cm^2^) were measured using a Professional Plus Model YSI multi‐parameter sonde (model 10,102,030; Yellow Springs Inc.) at the deepest point of each lake in June 2018 and June 2019. Secchi depth (m) was estimated by lowering a Secchi disk on the shaded side of boat. We collected water samples to quantify dissolved organic carbon (DOC; mg/L), total nitrogen (TN; mg/L), total phosphorous (TP; μg/L), chlorophyll *a* (chl‐*a*; μg/L), and dissolved calcium (Ca; mg/L). DOC, TN, TP, and chl‐*a* samples were analyzed at the GRIL‐Université du Québec à Montréal (UQAM) analytical laboratory. DOC concentrations of 0.45 μm filtered samples (surfactant‐free membrane filters) were measured after acidification (phosphoric acid 5%) followed by sodium persulfate oxidation using a 1010 TOC analyzer (O.I. Analytical, College Station, TX, USA). The absorption at 440 nm (CDOM), used as a measure of watercolor, was measured on water samples with a 2 cm quartz cuve in a BiochromUltrospec® 2100 pro spectrofluorometer (Cuthbert & del Giorgio, 1992). Total Nitrogen (TN) was analyzed with a continuous flow analyzer (OI Analytical Flow Solution 3100 ©) using an alkaline persulfate digestion method, coupled with a cadmium reactor, following a standard protocol (Patton & Kryskalla, 2003). Total phosphorus (TP) was measured spectrophotometrically on the same machine by the molybdenum blue method after persulfate digestion (Griesbach & Peters, 1991). Chl‐a was quantified by passing samples through glass fiber filters (Whatman GF/F), extraction of the Chl‐a in hot ethanol, and measuring the chlorophyll spectrophotometrically on a “BiochromUltrospec” 2100 pro with a 10‐cm quartz cuvette (Sartory & Grobelaar, 1984; Winterman & de Mots, 1965). Water calcium samples were analyzed with a Thermo ICAP‐6300 Inductively Coupled Argon Plasma—Optical Emission Spectrometer (ICP‐OES) following protocols described by US EPA (1994) at the University of Alberta Biogeochemical Analytical Service Laboratory (U of A—BASL; Edmonton, Alberta, Canada).

## APPENDIX 4 DETAILS OF THE IMPLEMENTATION

In a series of capture “waves,” fish were caught in unbaited minnow traps from the eight source lakes and transported by car in large coolers to a cabin in Kenai, where we had set up temporary fish housing. On arrival, the fish were moved into large Tupperware bins labeled by source lake and bubbled with air and occasionally iced (with sealed ice packs) to help combat unseasonably warm temperatures toward the end of the experiment. The fish were not fed in order to maintain water quality high and because we were aiming to keep them onsite for only short periods of time. Water changes were performed multiple times through the day using both filtered well water and water from local lakes.

The processing protocol involved lightly anesthetizing individual stickleback with diluted MS‐222 (tricaine) so that they did not move while being photographed. Each fish was then photographed with an individual label under standard lighting in shallow water. After the photograph, a small piece of caudal fin tissue was clipped and preserved with the individual label in 95% EtOH. The fish were then allowed to recover and were placed into new “bins” for each restoration lake containing their respective mixed pools (benthic, limnetic, or mixed [the last for Loon Lake]).

To ensure that fish were not held for too long, we planned waves of introductions to the lakes. That is, we would finish processing a number of individuals for a given pool, introduce this wave into the restoration lakes, and then start on another wave for another introduction time, and so on until we had introduced the target number of fish. We also strove to introduce benthic and limnetic pools into their respective pair of restoration lakes at approximately the same time and rate. That is, each “wave” of introductions would be matched for benthic‐pool and limnetic‐pool restoration lakes.

In the first introduction phase, the eight restoration lakes (four receiving the benthic pool and four receiving the limnetic pool) each received approximately 75 fish from each of the four source lakes. With the second introduction phase, we decided to “fill” (with 25 more fish from each source lake) the smallest two lakes (Fred's and Leisure Pond) that were planned to receive 100 fish from each source lake. From there, we filled the next largest lakes (Ranchero Lake and CC Lake), each receiving 200 fish from each source lake. So, by the end of the second introduction phase, we had completed introductions for four of the eight lakes. With the third introduction phase, we split fish between the remaining two lakes of each type equally. We also started building the pools for the mixed population being introduced to Loon Lake, which was the last introduction to be completed.

Any fish that died after processing, but before mixing into the pools, were counted and replaced with equal numbers of fish from the source lakes—thus ensuring the target number of live fish was released. The final realized numbers of released fish from each source lake into each recipient lake is shown in Table 4, wherein the largest deviation from the original design is also explained in the caption. Some deviations will occur from the estimated numbers of fish per source lake into each restoration lake because we did not know the source lakes of the fish that died post‐processing but before release (because they were held together as a pool). Genotyping the dead fish will resolve this issue.
